# Wind Profiling in the Lower Atmosphere from Wind-Induced Perturbations to Multirotor UAS

**DOI:** 10.3390/s20051341

**Published:** 2020-02-29

**Authors:** Javier González-Rocha, Stephan F. J. De Wekker, Shane D. Ross, Craig A. Woolsey

**Affiliations:** 1 Department of Aerospace and Ocean Engineering, Virginia Tech, Blacksburg, VA 24060, USA; sdross@vt.edu (S.D.R.); cwoolsey@vt.edu (C.A.W.); 2 Department of Environmental Sciences, University of Virginia, Charlottesville, VA 22903, USA; dewekker@virginia.edu

**Keywords:** unmanned aircraft systems, system identification, wind estimation, multi-rotor, drone, atmospheric science, wind profile, boundary layer meteorology

## Abstract

We present a model-based approach to estimate the vertical profile of horizontal wind velocity components using motion perturbations of a multirotor unmanned aircraft system (UAS) in both hovering and steady ascending flight. The state estimation framework employed for wind estimation was adapted to a set of closed-loop rigid body models identified for an off-the-shelf quadrotor. The quadrotor models used for wind estimation were characterized for hovering and steady ascending flight conditions ranging between 0 and 2 m/s. The closed-loop models were obtained using system identification algorithms to determine model structures and estimate model parameters. The wind measurement method was validated experimentally above the Virginia Tech Kentland Experimental Aircraft Systems Laboratory by comparing quadrotor and independent sensor measurements from a sonic anemometer and two SoDAR instruments. Comparison results demonstrated quadrotor wind estimation in close agreement with the independent wind velocity measurements. However, horizontal wind velocity profiles were difficult to validate using time-synchronized SoDAR measurements. Analysis of the noise intensity and signal-to-noise ratio of the SoDARs proved that close-proximity quadrotor operations can corrupt wind measurement from SoDARs, which has not previously been reported.

## 1. Introduction

Measuring wind velocity near the Earth’s surface is critical to understanding the surface-atmosphere interactions driving the dynamic state of the atmospheric boundary layer (ABL). How the ABL evolves with space and time impacts public health and safety [1,2,3,4,5,6], transport of air pollutants, pollen and spores [7,8,9,10], wind power supply to smart grid systems [11,12,13,14,15], forecast of local weather [3,4,5,6,16], air traffic control at airports [17,18,19,20], the spread and management of wildfires [21,22,23,24], and emissions mitigation of greenhouse gases [25,26,27,28,29]. Therefore, accurately characterizing the dynamic state of the ABL over micro- and mesoscale domains is important [3,16,30,31]. However, observations of wind velocity at high spatial resolution are difficult to attain due to the cost and limited mobility of conventional atmospheric sensing technology.

Early work to address the existing gap of atmospheric wind measurements and atmospheric parameters such as atmospheric pressure, air temperature, and relative humidity (PTH) involved fixed-wing aircraft for their predictable dynamics, payload capacity, and flight endurance. Approaches to measuring wind with fixed wing aircraft consist of direct and indirect measurements of wind velocity. Direct methods have combined inertial and air-relative velocity measurements from GPS and a Pitot tube or multi-hole air data system to infer wind velocity using the wind triangle relationship [32,33]. Indirect methods, on the other hand, have exploited GPS and IMU measurements along with vehicle dynamic models to estimate wind velocity employing state estimation algorithms [34,35]. A comprehensive review of established direct and indirect methods for inferring wind with fixed-wing aircraft is presented in [36]. However, despite the advantages of operating fixed wing aircraft over open space, safely maneuvering in urban and complex environments to measure wind speed and PTH variables remains a challenge.

More recently, multirotor UAS have become popular for direct and indirect measurements of atmospheric wind and PTH variables within the ABL. Multirotor UAS are mobile, portable, low cost, and easy to operate over complex and urban environments where it is prohibitively difficult for conventional atmospheric sensors or fixed-wind aircraft. Direct methods involve measuring wind velocity from an on-board flow sensor, which include various types of anemometers [37,38,39,40,41] and air data systems integrated with Pitot tubes or multi-hole probes [42,43]. The choice of sensor for wind sensing depends on size and power requirement of the sensor, as well as the airframe configuration and payload capacity of the multirotor UAS. Indirect methods, on the other hand, estimate wind velocity from wind induced perturbations to the aircraft motion and do not require an onboard airflow sensor. Conventional model-based approaches to wind estimation have involved kinematic [44,45], point mass [1,39,46,47,48], and rigid body models [1,49] of control-augmented quadrotor dynamics, which characterize how a quadrotor responds to disturbances under feedback stabilization. A comparison of all three vehicle motion models has demonstrated that both the accuracy and bandwidth of wind estimates increases with model fidelity [1], which has made model-based wind sensing inside the ABL more useful and reliable. Especially in scenarios where direct measurements are impossible, i.e., when the aircraft is very small or when the aircraft is intended for some other purpose and is not equipped to measure the wind.

To date, model-based wind estimation approaches accounting for vehicle dynamics have mostly incorporated models appropriate for *hovering* flight [1,46,47,48,49,50]. Measuring wind velocity only while hovering limits the efficiency of multirotor aircraft to sample the lower atmosphere. The limited effectiveness of stationary sampling is largely due to the endurance of multirotor aircraft, which is typically less than 20 min. Many applications of multirotor UAS wind sensing require atmospheric sampling over horizontal and vertical distances. Therefore, there is a need to develop wind estimation algorithms that allow for movement of the multirotor UAS while accurately measuring wind velocity inside the ABL.

This paper presents the validation of a model-based method for estimating vertical profiles of the horizontal wind velocity employing wind measurements from a sonic anemometer and SoDAR (Sound Detection And Ranging) wind profilers. The model-based method that is validated, referred as the *dynamic rigid body wind profiling* method or *DRBWindPro* method, is the extension presented in [2] of the wind sensing algorithm described in [1] to infer wind velocity using a dynamic rigid body model for hovering flight. The extension of the wind sensing algorithm incorporates dynamic rigid body models characterized from system identification for equilibrium flight conditions corresponding to steady ascent rates ranging from 0 to 2 m/s. The models from system identification were used to estimate the wind velocity in the vicinity of ground-based in situ and remote atmospheric sensors. Quadrotor wind estimates and wind measurements from ground-based atmospheric sensors were then compared to determine the accuracy of the DRBWindPro method.

The organization of this paper is as follows. Section 2 introduces materials and methods used for model-based wind estimation. This section includes the formulation of aircraft dynamics, system identification of aircraft models, and the design of a state observer for wind estimation. The ground-based wind measurement methods are described in Section 3. In Section 4, results from system identification experiments and comparison of multirotor wind velocity measurements with ground-based measurements are presented. Section 5 presents a thorough discussion of results from system identification and from comparing multirotor and ground-based wind measurements. Finally, a summary of findings and future work to extend the utility of multirotor UAS for wind sensing are presented in Section 6.

## 2. Materials and Methods

### 2.1. Modeling Framework

The equations of motion for a control-augmented (i.e., feedback-stabilized) quadrotor can be expressed as a system of first-order, nonlinear, time-invariant ordinary differential equations [1,2]:

(1) $$\dot{x}=f\left(x,u,w\left(t,x\right)\right),\;x\left(t_{0}\right)=x_{0}$$

relating the rate of change $\dot{x}$ of the vehicle’s 12-dimensional state x (i.e., position, attitude, velocity, and angular velocity), to the state itself, the control inputs u, and wind disturbances w(t,x) varying over time and space. Moreover, when the aircraft motion is modeled as a small perturbation from some equilibrium flight condition that corresponds to a constant vertical ascent speed denoted by $V_{z_{\mathrm{eq}}}$, the nonlinear dynamics describing the control-augmented motion of the quadrotor is well approximated by a linear model. As a result, one may infer wind velocity from wind-induced motion perturbations to a quadrotor employing estimation theory developed for linear systems.

Linear approximations of quadrotor dynamics for wind estimation are considered in this study for hovering and steady-ascending motions satisfying trim flight conditions. For a quadrotor, trim flight conditions are satisfied when both translational rates v and rotational rates ω remain constant over time, i.e., $\dot{v}\equiv 0$ and $\dot{\omega }\equiv 0$. Linear approximations of quadrotor dynamics for hovering and ascending flight are in the form,

(2) $$\frac{d}{dt}\tilde{x}=A\tilde{x}+B\tilde{u}+\Gamma w,$$

where the vectors $\tilde{x}=x-x_{\mathrm{eq}}$ and $\tilde{u}=u-u_{\mathrm{eq}}$ denote, respectively, small deviations in the state and input vectors from their steady-state values. Additionally, the state matrix $A\in R^{12\times 12}$ models unforced dynamics, the input matrix $B\in R^{12\times 4}$ characterizes applied forcing, and the disturbance matrix $\Gamma \in R^{12\times 3}$ captures wind-induced perturbations. This model form is used to estimate the horizontal component wind velocity at different steady motion conditions ${V_{z}}_{\mathrm{eq}}$.

### 2.2. Aircraft System Identification

**Figure 1:**
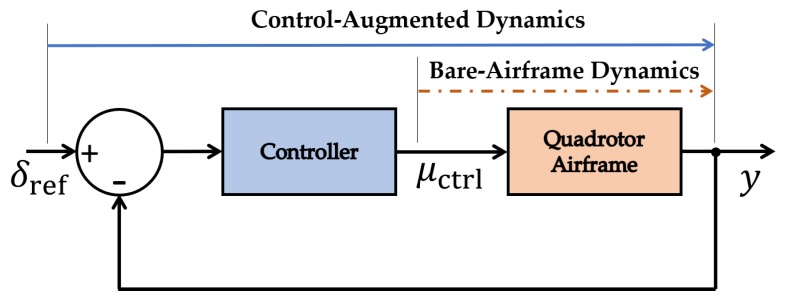
A schematic of input-output signals for closed-loop and open-loop mappings.

Aircraft system identification is used to characterize the state and input matrices A and B for a quadrotor flying in still air conditions (i.e., w(x,t)≈0 m/s). In general, this modeling approach is a multi-faceted process that relies on input-output flight test data to characterize bare-airframe or control-augmented dynamic models for an aircraft, depending on application. Figure 1 shows a schematic of the inputs u and outputs y used to identify bare-airframe and control-augmented models. A bare-airframe model, assuming actuator dynamics to be negligible, is identified using control signals from the flight controller $\mu_{\mathrm{ctrl}}$ and the vehicle’s measured dynamic response y. A control-augmented model, alternatively, is identified using the reference signal $\delta_{r}$ from pilot-induced joystick commands and the vehicle’s measured dynamic response y. Which model is identified depends on its application. For wind estimation purposes with an off-the-shelf quadrotor, we use the latter because it does not require knowledge of the onboard flight controller architecture.

**Figure 2:**
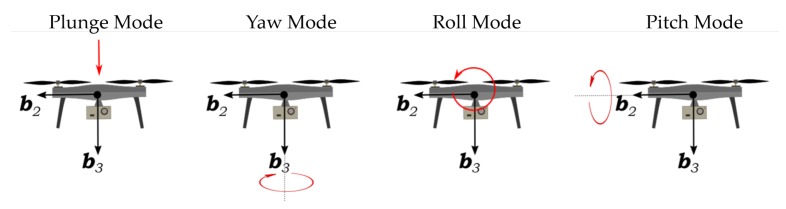
Quadrotor plunge, yaw, roll, and pitch modes.

The quadrotor models from system identification are for steady-state equilibrium flight conditions corresponding to the hovering and steady ascending flight: ${V_{z}}_{\mathrm{eq}}=\left\{0.0,0.5,1.0,1.5,2.0\right\}$ m/s. The identification of each model involved separately determining four sub-models that describe the plunge, yaw, roll, and pitch dynamics of the quadrotor; see Figure 2. In this process, stepwise regression was used first to determine the parameter structure of each model. Results from stepwise regression were then used to estimate model parameters using an output error algorithm. This approach to system identification was used to minimize the set of parameters being estimated at one time and to avoid overparameterized models.

#### 2.2.1. Multirotor UAS Platform

**Figure 3:**
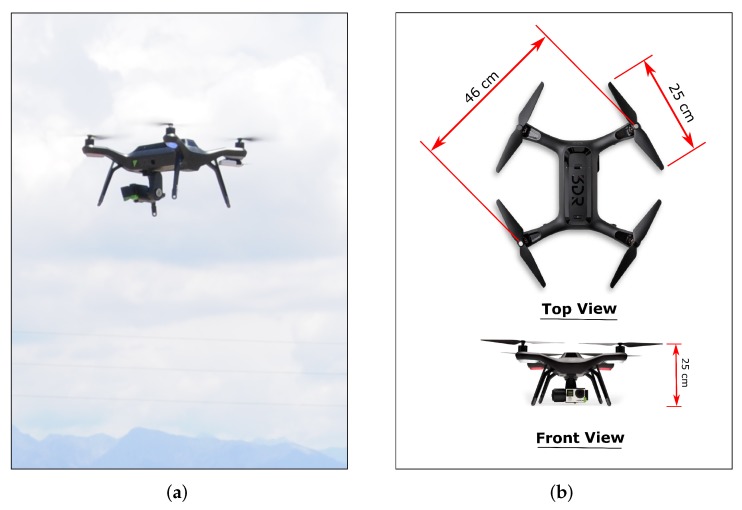
(**a**) The multirotor UAS employed for validation of model-based wind estimation along with (**b**) dimensions.

The multirotor UAS used to measure the wind velocity is an off-the-shelf 3DR Solo quadrotor shown in Figure 3. This aircraft is 25 cm tall with a 46 cm diagonal between motor shafts. Fully equipped with a lithium polymer battery pack and a 3-axis camera gimbal, the quadrotor weighs 1.5 kg and has a payload capacity of 0.5 kg. The propellers used with the quadrotor are a Master Airscrew 10×4.5 propeller set. The quadrotor’s autopilot is a Pixhawk 2.1 Green Cube manufactured by ProfiCNC. The autopilot operates using open-source Arducopter firmware and is compatible with MissionPlanner and Solex telemetry software. On board the Pixhawk 2.1 Green Cube are the sensors listed in Table 1 that are part of the autopilot’s attitude and heading reference system (AHRS).

#### 2.2.2. System Identification Flight Testing

System identification flight experiments were conducted outdoors in an open field adjacent to the Virginia Tech Kentland Experimental Aircraft Systems (KEAS) Laboratory to characterize quadrotor linear models for wind estimation. The flight experiments were designed to identify models approximating the quadrotor dynamics about the equilibrium flight conditions corresponding to ${V_{z}}_{\mathrm{eq}}=\left\{0.0,0.5,1.0,1.5,2.0\right\}$ m/s. The experiments required exciting the aircraft from each flight equilibrium in calm atmospheric conditions (i.e., w(x,t)≈0 m/s) to minimize the impact of exogenous excitations on the system identification process. The input-output measurements used for system identification consisted of pilot-induced, sinusoidal joystick commands and the vehicle’s measured dynamic response.

The system identification experiments were performed in two parts. A first set of experiments were performed to identify the quadrotor’s hovering flight dynamics. This required exciting from equilibrium flight the quadrotor’s plunge, yaw, roll and pitch dynamics shown in Figure 2. A second set of experiments was conducted to identify quadrotor models for constant ascent rates varying between 0.5 and 2 m/s. This involved exciting the quadrotor’s roll and pitch dynamics from equilibrium flight conditions corresponding to ${V_{z}}_{\mathrm{eq}}>0$. For the latter case, the plunge and yaw dynamics of the quadrotor were assumed to be well approximated by models identified for hovering flight considering that the vehicle’s response to wind perturbations in steady-ascending flight is dominated by roll and pitch motions. Measurements from both sets of system identification experiments were then used to identify the model structures and parameter estimates approximating the quadrotor’s dynamics for all five operating conditions specified by $V_{z_{\mathrm{eq}}}$.

#### 2.2.3. Model Structure Determination

The parameter structure of each model was determined from input-output measurements employing the stepwise regression algorithm described in [51]. Using this approach, a set of postulated regressors, $\chi =\{\chi_{1},\chi_{2},\cdots ,\chi_{n}\}$ is tested one at a time to determine which ones significantly improve the fit of the model

(3) $$z\left(k\right)=a_{0}+\sum_{i=1}^{m}a_{i}\chi_{i}\left(k\right),\;k=1,2,\cdots ,N$$

where *z* is the quadrotor’s measured response, $a_{0}$ is the model bias, $a=\{a_{0},a_{1},\cdots ,a_{m}\}$ is the set of model coefficients associated with *m* regressors, and *N* is the sample size of measurements. How well each model structure fits the observed data as regressors are added or removed is determined using the $F_{0}$ statistic and coefficient of determination $R^{2}$ metrics. The $F_{0}$ statistic quantifies how much each regressor contributes to the fit of the model. The coefficient of determination quantifies how well the model output matches the measured data. Using both metrics, a total of four parameter structures were identified to characterize the quadrotor’s plunge, yaw, roll, and pitch dynamics.

#### 2.2.4. Parameter Estimation

The model structures determined from step-wise regression were used to initialize the estimation of model parameters using the output error algorithm described in [51]. The output error algorithm estimates model parameters using the output of the linear aircraft model described by Equation (2) in still air conditions and using the *N* sample points of measured flight data, which are assumed to be corrupted by sensor noise η. The model and measurements used by the output error method are summarized below:

(4) $$\begin{array}{ccc} \frac{d}{dt}\tilde{x} & = & A\tilde{x}+B\tilde{u},\;\tilde{x}\left(0\right)=x_{0} \end{array}$$

(5) $$\begin{array}{ccc} y & = & C\tilde{x}+D\tilde{u} \end{array}$$

(6) $$\begin{array}{ccc} z(k) & = & y(k)+\eta (k)\;k=1,2,\cdots ,N \end{array}$$

where y is the output vector, z is the measurement vector, C is the output matrix, and D is the feedthrough matrix. This formulation of the output error method assumes that the model being identified is free of process noise, making numerical propagation of state measurements possible. Moreover, output error parameter estimation assumes flight measurements to be corrupted with uncorrelated, zero-mean Gaussian noise $\eta \in N(0,R_{\mathrm{Cov}})$ such that the covariance matrix of measurement noise is diagonal,

$$\mathrm{Cov}\left(\eta \left(k\right)\right)=E\left[\eta \left(k\right)\eta^{T}\left(k\right)\right]=R_{\mathrm{Cov}}$$

Using this framework, parameter estimates are tuned iteratively while minimizing the cost function,

(7) $$J=\frac{1}{2}\sum_{i=1}^{N}{\left[y\left(k\right)-z\left(k\right)\right]}^{T}R_{\mathrm{Cov}}^{-1}\left[y\left(k\right)-z\left(k\right)\right]$$

which is the uncertainty-weighted residual between the model output and observation measurements.

Employing the output error approach, three sets of parameters were estimated and averaged to characterize quadrotor models for hovering and steady vertical ascent conditions. The quadrotor models characterized from averaged parameter estimates were validated using a separate flight test data set collected during system identification experiments.

#### 2.2.5. Model Validation

Linear models approximating steady-flight quadrotor dynamics were validated using input-output data collected separately during system identification flight experiments. The validation process for linear models involved comparing model outputs and state measurements corresponding to pilot-generated excitations using the root-mean-squared error (RMSE) metric:

$$RMSE=\sqrt{\frac{1}{N}\sum_{k=1}^{N}{\left(y\left(k\right)-z\left(k\right)\right)}^{2}}$$

where *y* is the model output, *z* is the state measurements, and *N* is the measurement sample size. In general, small RMSE values are indicative of accurate parameter estimates. Results from the RMSE quantification were used to assess the goodness of each model prior to designing a state observer for wind estimation.

### 2.3. Observer Synthesis

To synthesize observers for wind velocity estimation, the dynamic rigid body wind sensing method presented in [1,2] was adapted. Therefore, assuming absolute measurements from the GPS antenna and AHRS on board the quadrotor to be available, the output equation, as in [1,2], is of the form

$$y=I_{12}\tilde{x}+(\begin{array}{c} 0_{3}\\ 0_{3}\\ I_{3}\\ 0_{3} \end{array})w$$

where output measurements of translational velocity are the summation of both air-relative and wind velocity (with identity and zero matrices written in short notation, e.g., $I_{12}\in R^{12\times 12}$). We assume in this formulation that sensor noise in the output measurement is negligible, and is therefore not accounted for. The quadrotor’s output measurement and identified models were then used to formulate wind-augmented models for the set of operating conditions prescribed by ${V_{z}}_{\mathrm{eq}}$.

Wind velocity was estimated using the quadrotor models identified from system identification in a state observer framework. State observers were developed based on wind-augmented models corresponding to each of five equilibrium flight conditions. Each wind-augmented model is obtained by reformulating (2) such that the wind disturbance is part of the wind-augmented state vector: $x_{A}={\left[{\tilde{x}}^{T},w^{T}\right]}^{T}$. Here, as in [1,2], variations of wind velocity with respect to time were assumed to vary slowly relative to the dynamics of the quadrotor such that $\frac{d}{dt}w\approx 0$. Therefore, wind-augmented dynamic models corresponding to each flight equilibrium were defined as follows:

(8) $$\frac{d}{dt}x_{A}=\underset{A_{A}}{\underset{︸}{\left(\begin{array}{cc} A & \Gamma \\ 0_{3\times 12} & 0_{3} \end{array}\right)}}x_{A}+\underset{B_{A}}{\underset{︸}{\left(\begin{array}{c} B\\ 0_{3\times 4} \end{array}\right)}}\tilde{u}\;y=\underset{C_{A}}{\underset{︸}{\left(\begin{array}{ccccc} I_{3} & 0_{3} & 0_{3} & 0_{3} & 0_{3}\\ 0_{3} & I_{3} & 0_{3} & 0_{3} & 0_{3}\\ 0_{3} & 0_{3} & I_{3} & 0_{3} & I_{3}\\ 0_{3} & 0_{3} & 0_{3} & I_{3} & 0_{3} \end{array}\right)}}x_{A}$$

where $A_{A}\in R^{15\times 15}$ is the wind-augmented state matrix, $B_{A}\in R^{15\times 4}$ is the wind-augmented input matrix, and $C_{A}\in R^{12\times 15}$ is the wind-augmented output model.

To verify the observability of the augmented dynamic model, an observability analysis was conducted to determine if wind estimates can be realized from the model and output measurements. The system is observable if and only if the observability matrix defined below is column-wise full rank.

$$O\left(C_{A},A_{A}\right)=(\begin{array}{c} C_{A}\\ C_{A}A_{A}\\ \vdots \end{array})=(\begin{array}{ccccc} I_{3} & 0_{3} & 0_{3} & 0_{3} & 0_{3}\\ 0_{3} & I_{3} & 0_{3} & 0_{3} & 0_{3}\\ 0_{3} & 0_{3} & I_{3} & 0_{3} & I_{3}\\ 0_{3} & 0_{3} & 0_{3} & I_{3} & 0_{3}\\ 0_{3} & G_{w} & I_{3} & 0_{3} & I_{3}\\ 0_{3} & 0_{3} & 0_{3} & I_{3} & 0_{3}\\ 0_{3} & G_{g} & -d_{w}e_{3}e_{3}^{T} & 0_{3} & 0_{3}\\ 0_{3} & 0_{3} & D_{m_{v}} & D_{m_{\omega }} & 0_{3}\\ \vdots & \vdots & \vdots & \vdots & \vdots \end{array})$$

The analysis shows that the observability matrix is full rank, i.e., $\mathrm{rank}\left[O(C_{A},A_{A})\right]=15$. Therefore, computing a suitable observer gain matrix $G_{O}$, state estimates of the following observer will converge to the state of the system (8)

(9) $$\frac{d}{dt}\;\hat{x_{A}}=A_{A}\hat{x_{A}}+B_{A}u+G_{O}\left(y-C_{A}\hat{x_{A}}\right)$$

Because the augmented state vector includes the wind velocity, it follows that the state estimator (9) provides a convergent estimate of w, provided the underlying assumptions hold (e.g., small perturbations from the nominal state). Moreover, in the implementation of this framework for steady vertical-ascent wind estimation, the appropriate set of model parameters was switched manually offline before processing quadrotor flight measurements.

## 3. Experimental Validation of Wind Estimates

### 3.1. Field Experiment Setup

Field experiments were performed at the KEAS Laboratory on June 5th, 2018 from 15:00 to 20:30 EDT to validate horizontal wind velocity estimates from quadrotor hover and vertical steady ascent conditions: ${V_{z}}_{\mathrm{eq}}=\left\{0,0.5,1.0,1.5,2.0\right\}$ m/s. Originally, we intended to validate quadrotor wind estimates using observations from ground-based sensors and a small solid-state sonic anemometer mounted on board a separate multirotor UAS. However, we were unable to use the solid-state anemometer due to a hardware malfunction. Thus, the accuracy of the model-based wind estimates was examined using measurements from ground-based sensors only.

**Figure 4:**
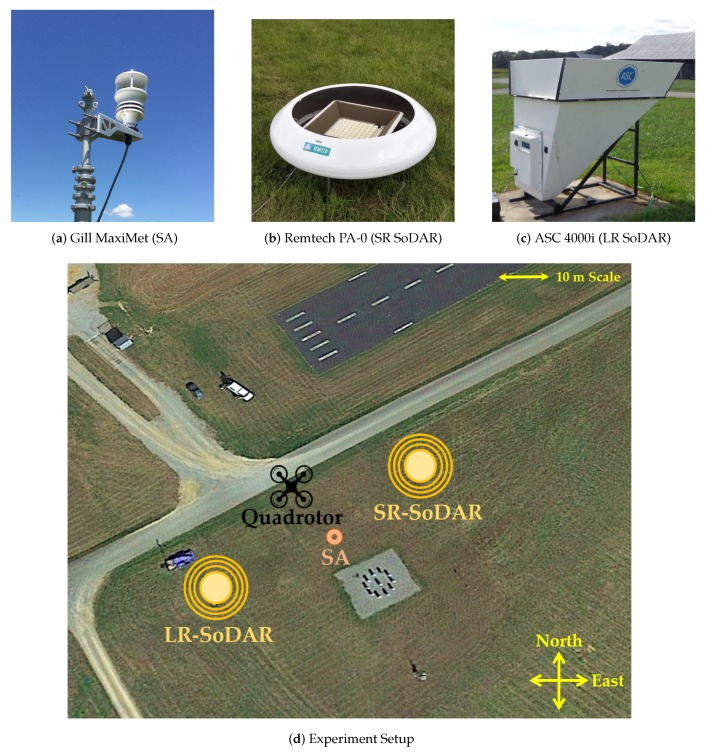
(**a**) The Gill MaxiMet sonic anemometer used to measure wind velocity 10 m AGL. (**b**) The Remtech PA-0 sensor (SR SoDAR) used to measure wind velocity from 10 to 120 m AGL. (**c**) The ASC 4000i sensor (LR SoDAR) used to measure wind velocity from 30 to 120 m AGL. (**d**) The experiment setup used to validate quadrotor wind estimates from 10 to 120 m AGL.

The ground-based sensors that were used to validate quadrotor estimates of horizontal wind velocity consist of the sonic anemometer and SoDAR wind profilers found in Figure 4. The Gill MaxiMet sonic anemometer (SA) shown in Figure 3a and the Remtech PA-0 SoDAR (SR-SoDAR) shown in Figure 4b were used to validate quadrotor wind estimates at 10 m AGL. Alternatively, quadrotor profiles of horizontal wind velocity were validated using both the Remtech PA-0 and the ASC4000i SoDAR (LR-SoDAR) shown in Figure 4c. The configuration of the three sensors relative to quadrotor operations is shown in Figure 4d. Additionally, the performance envelope of each sensor is found in Table 2. Using this sensor configuration quadrotor wind velocity estimates were validated after processing observations from independent sensors.

It is also important to note that our focus during validation experiments laid primarily on estimating the horizontal component of wind velocity while hovering or profiling due to constraints driven by science objectives, the flight envelope of the quadrotor, and experiment setup. Constant-rate vertical profiling with multirotor UAS is already used to measure PTH within the ABL [3,16], and while it is possible for a multirotor aircraft to measure PTH in descent [31], steady descent rates are only realizable at very low speeds. Therefore, wind estimation in steady vertical descent may be inefficient for model-based wind profiling. Moreover, while we are certainly interested in horizontal profiling and, more generally, wind profiling for arbitrary steady motions, the independent sensors (i.e., SoDARs) that were used in this study only provide vertical wind profiles. Thus, we were only able to validate vertical profile measurements.

### 3.2. Comparison with Ground-Based Observations

We also determine the limitations of wind validation experiments by comparing differences across ground truth observations at different heights. When observation differences are small, we can validate quadrotor wind estimates reliably. This is because observations collected at distinct locations will not be representative of the wind field measured by the quadrotor in high variability conditions. To compare independent wind observation at different heights, sonic anemometer and LR-SoDAR observations recorded every 1 and 30 s, respectively, were averaged to match the 300-s sampling period of the SR-SoDAR. Differences across sensor observation were then characterized using the mean difference error (MDE) and RMSE metrics at 10, 60 and 110 m AGL. Results from the comparisons were used to assess the accuracy of quadrotor wind estimates for different flight regimes.

## 4. Results

### 4.1. System Identification

#### 4.1.1. Model Structure Determination

The quadrotor flight dynamic model for hovering and steady ascending flight is decomposed into four sub-models that describe plunging, yawing, rolling, and pitching motion. Table 3 shows all four model forms and associated parameters. The plunge model is a system of two first-order ordinary differential equations parameterized by propulsive and damping parameters. The yaw model is a system of two first-order ordinary differential equations with rotational damping and stiffness parameters. Finally, the roll and pitch models are systems of four first-order ordinary differential equations.

#### 4.1.2. Parameter Estimation

Three sets of quadrotor model parameters were estimated for each of five equilibrium flight conditions. Each set of parameters was estimated using the model structures determined from step-wise regression and the output error algorithm described in Section 2.2. Model parameters for the plunge, yaw, roll, and pitch model structures were first estimated for hovering flight conditions (i.e., v=0 and ω=0). Subsequently, roll and pitch model parameters were estimated for constant vertical ascent flight conditions varying from 0.5 to 2.0 m/s. We assume the plunge and yaw model parameters to be invariant with vertical ascent rate. Model parameter estimates for each flight equilibrium were averaged to obtain nominal models for wind estimation. Averaged parameter estimates and standard error (SE) values for plunge and yaw models are listed in Table 4. Additionally, averaged roll and pitch model parameters and standard error values are listed for hovering and ascending flight conditions in Table 5 and Table 6, respectively.

**Figure 5:**
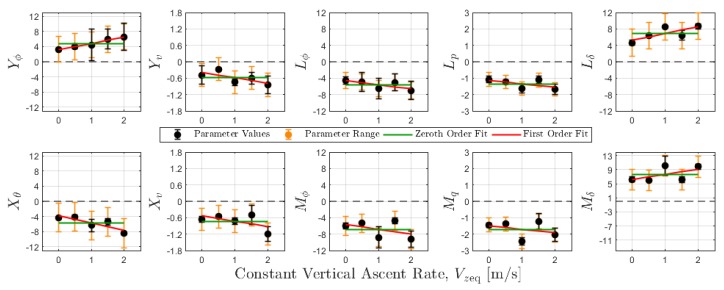
Roll and pitch model parameter estimates corresponding to vertical constant ascent rates ${V_{z}}_{\mathrm{eq}}=\left\{0.0,0.5,1.0,1.5,2.0\right\}$ m/s.

The dependence on vertical ascent rate was also characterized for roll and pitch quadrotor parameters. Results from this characterization are shown in Figure 5 where roll and pitch model parameters are plotted as a function of ascent rate. Each parameter estimate appears with absolute error bars, colored in black, representing the range of estimates obtained from the three experimental data sets. Orange-colored bars were also included to denote minimum and maximum values across all five ascent rates. Zeroth- and first-order polynomials were fit to the parameter estimates as a function of ascent rate. The first-order fit, on the other hand, characterizes the trend in parameter values with respect to ascent rate. Note that only a subset of parameters exhibit clear trends with respect to ascent rate. It is possible that these local, small-perturbation models do exhibit high sensitivity to ascent rate, as suggested by Figure 5. If so, then these results may suggest flight regimes to be avoided when estimating wind velocity from platform motion; regions of high parameter sensitivity may produce less accurate wind estimates.

For the aircraft and dynamic model considered here, the parameters vary less at lower ascent rates (0.5 m/s or less). Thus, one might expect more accurate wind measurements during slower climbs. It is possible, however, that the variation in parameter estimates is an artifact of the data collection method for system identification. At higher climb rates, it is more difficult to manually generate the rich and precisely timed excitation signals needed for model identification. An automated approach to system identification may improve the repeatability of parameter estimates.

#### 4.1.3. Model Validation

**Figure 6:**
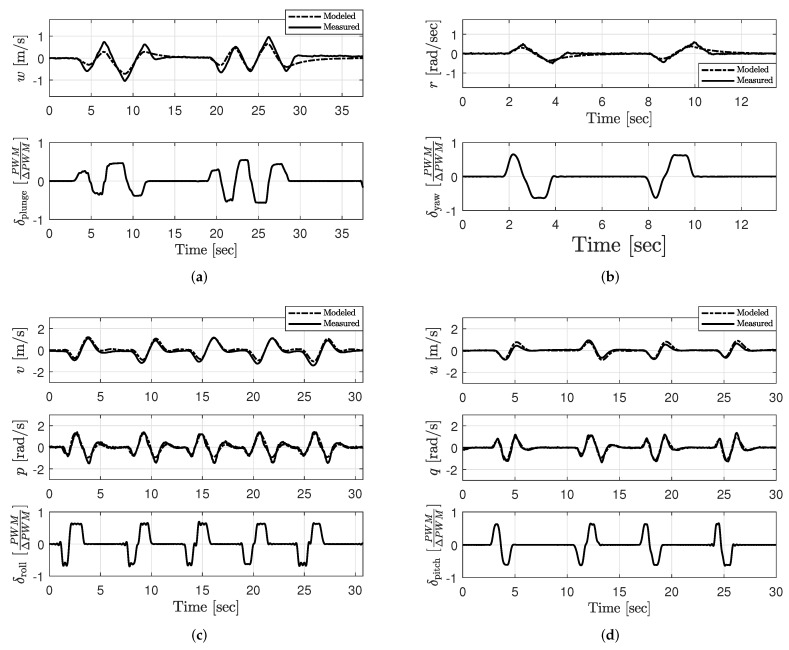
Validation of the (**a**) plunge, (**b**) yaw, (**c**) roll, and (**d**) pitch models identified for quadrotor hovering flight.

Models characterized from step-wise regression and output error methods were validated by comparing the model output and aircraft’s response to an excitation input. Agreement between the model output and measured response is compared using the RMSE metric discussed in Section 2.2.5. Results from this validation are shown in Figure 6 for the plunge, yaw, roll, and pitch models characterized for hovering flight ${V_{z}}_{\mathrm{eq}}=0$. Results from the RMSE assessment for the plunge and yaw models are shown in Table 7. The RMSE results for the pitch and roll models associated constant vertical ascent rates ranging between 0 and 2 m/s are also shown in Table 7.

### 4.2. Comparison of Wind Velocity Measurements

#### 4.2.1. Sonic Anemometer and SoDAR Comparison

**Figure 7:**
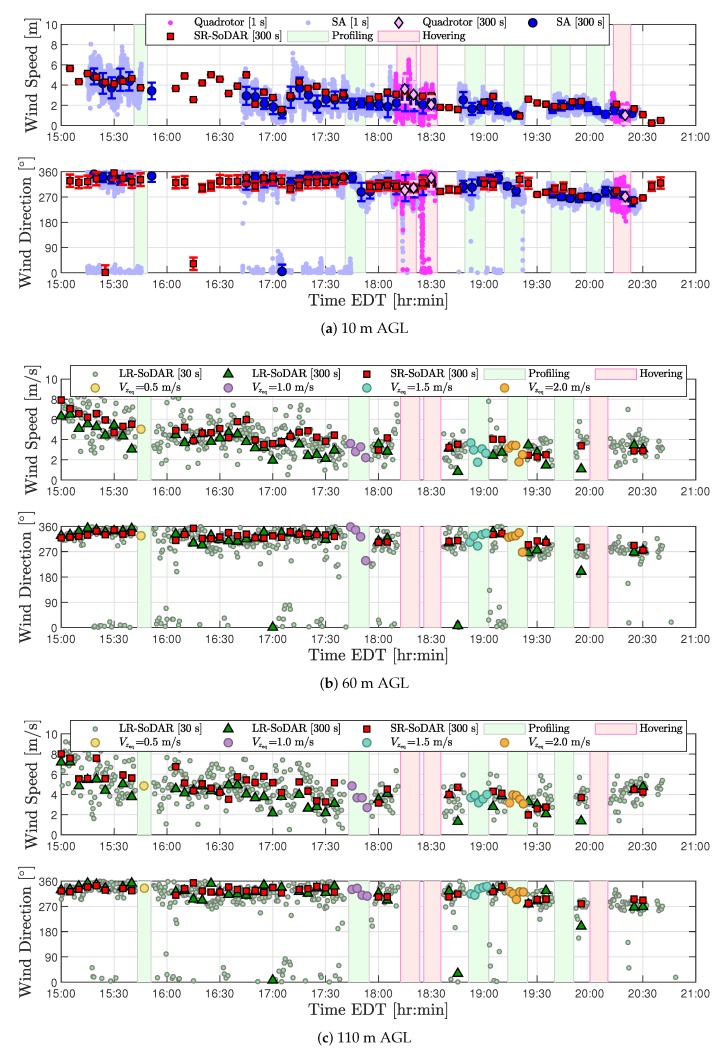
Comparison of wind observations collected from the quadrotor and independent sensors at (**a**) 10 m AGL, (**b**) 60 m AGL, and (**c**) 110 m AGL from 15:00 to 20:30 EDT on 5 June 2018.

The difference across sonic anemometer and SR-SoDAR wind measurements was characterized from 15:00 to 20:30 EDT to assess the spatial variability of wind at 10 m AGL. Based on 300-s averaged measurements from the sonic anemometer, prevailing wind conditions during validation experiments were from the northwest with wind speeds ranging from 1.2 to 4.0 m/s (see Figure 7a). As shown in Table 8, the MDE and RMSE values of wind speed observations were measured to be 0.7 m/s and 1.0 m/s, respectively. Wind direction MDE and RMSE values were measured as $32^{\circ }$ and $100^{\circ }$. Therefore, the difference across the spatial separation of ground truth measurements used to validate quadrotor wind estimates at 10 m AGL is relatively small.

#### 4.2.2. SoDAR Comparison

Wind observations from the LR- and SR-SoDAR were compared from 15:00 to 20:30 EDT at 60 and 110 m AGL to assess the spatial variability of wind conditions during quadrotor wind profiling operations. The prevailing wind conditions as reported by the SR-SoDAR were from northwest with wind speeds ranging from 2.3 to 7.9 m/s at 60 m AGL and from 2.0 to 8.0 m/s at 110 m AGL. Wind observations from SR- and LR-SoDAR at 60 and 110 m AGL are shown in Figure 7c,d, respectively. As reported in Table 9, the maximum MDE and RMSE for wind speed and wind direction were observed at 110 m AGL. The MDE and RMSE of wind speed observations at 110 m AGL were found to be −0.9 m/s and 1.4 m/s, respectively. Wind direction MDE and RMSE values, on the other hand, were measured to be $0^{\circ }$ and $26^{\circ }$, respectively. Thus, spatial wind variations were also observed to be small at higher altitudes.

#### 4.2.3. Validation of Quadrotor Wind Estimates

Wind estimates from three quadrotor flights hovering at 10 m AGL between 18:00 and 20:30 were compared to sonic anemometer and SR-SoDAR wind observations. Results from the comparison are shown in Figure 7a, where the time lapse of each quadrotor flight is denoted with a rose-colored vertical band. How well quadrotor and ground-based wind measurements compared is reported in Table 10 using the MDE metric. The average of wind speed and wind direction of absolute MDE values of quadrotor wind speed estimates were found to be 0.6 m/s and 0.5 m/s relative to sonic anemometer and SR-SoDAR observations. The average absolute MDE values for quadrotor wind direction estimates relative to the sonic anemometer and SR-SoDAR were found to be 14${}^{\circ }$ and 10${}^{\circ }$ relative to sonic anemometer and SR-SoDAR measurements, as well. Therefore, quadrotor wind estimates from hovering flights were assessed to have an accuracy comparable to that of conventional ground-based wind sensors.

In contrast to assessing the accuracy of wind estimates at 10 m AGL, validating quadrotor wind profiles ascending vertically at various steady rates proved to be more involved. Results from a two-part assessment found in Appendix A revealed that quadrotor profiling operations corrupt ground-truth SoDAR observations. Consequently, making time-synchronized comparisons of quadrotor and SoDAR wind measurements for validation purposes was not possible for an accurate assessment of quadrotor wind estimates. To circumvent corrupted wind observations from SoDARs, quadrotor wind profiles were validated using SoDAR measurements collected before and after quadrotor operation as well as linearly-interpolated wind profiles.

**Figure 8:**
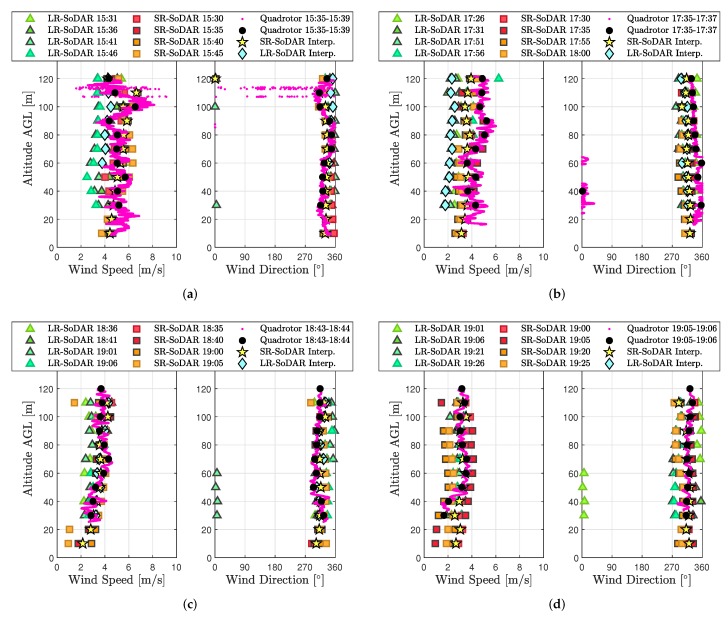
Comparison of wind speed and wind direction profiles from SoDAR and the quadrotor ascending vertically from 10 to 120 m AGL at (**a**) 0.5 m/s, (**b**) 1 m/s, (**c**) 1.5 m/s, and (**d**) 2 m/s.

**Figure A6:**
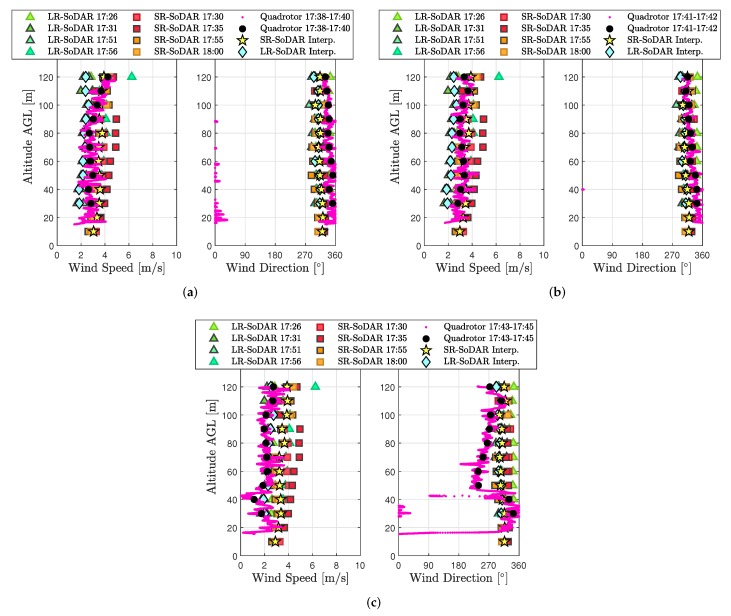
Comparison of wind speed and wind direction profiles from the SoDAR and quadrotor ascending vertically at 1.0 m/s between 10 and 120 m AGL from (**a**) 17:38 to 17:40 EDT, (**b**) 17:41 to 17:42 EDT, and (**c**) 17:43 to 17:45 EDT.

**Figure A7:**
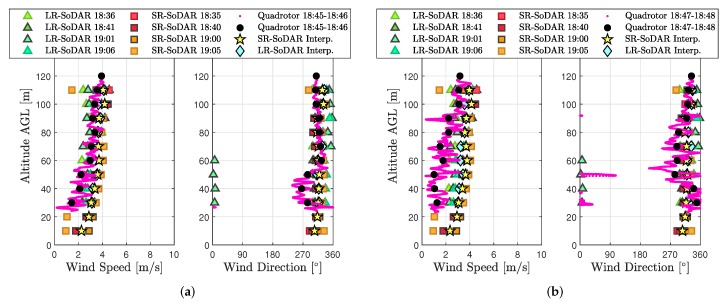

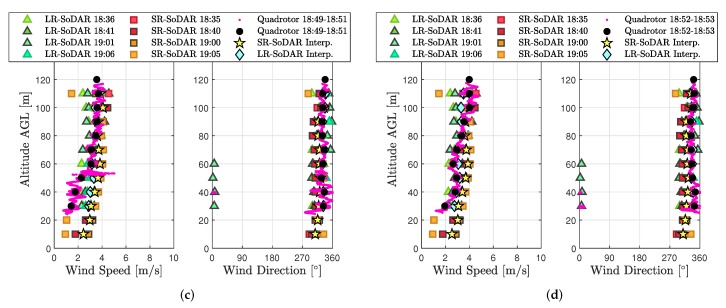
Comparison of wind speed and wind direction profiles from the SoDAR and quadrotor ascending vertically at 1.5 m/s between 10 and 120 m AGL from (**a**) 18:45 to 18:46 EDT, (**b**) 18:47 to 18:48 EDT, (**c**) 18:49 to 18:51 EDT, and (**d**) 18:52 to 18:53 EDT.

**Figure A8:**
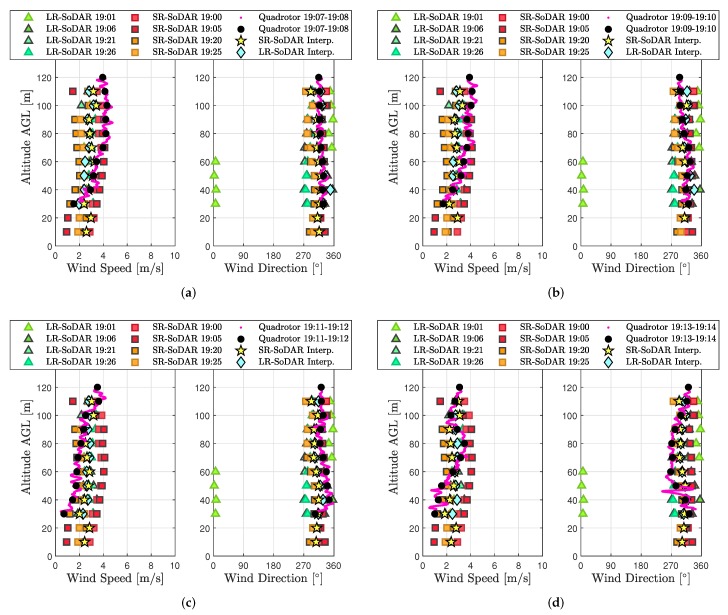
Comparison of wind speed and wind direction profiles from the SoDAR and quadrotor ascending vertically at 2.0 m/s between 10 and 120 m AGL from (**a**) 19:07 to 19:08 EDT, (**b**) 19:09 to 19:10 EDT, (**c**) 19:11 to 19:12 EDT, and (**d**) 19:13 to 19:14 EDT.

In total, four sets of quadrotor wind estimates corresponding to ${V_{z}}_{\mathrm{eq}}=\left\{0.5,1.0,1.5,2.0\right\}$ were compared to SoDAR wind observations to validate model-based wind profiling. Results from the comparisons shown in Figure 8c (and in Figure A7a,c,d of Appendix B) demonstrate quadrotor, SoDAR, and interpolated wind profiles to agree most accurately between 18:40 and 19:01 EDT, when wind variability across the sampling domain was observed to be the lowest. Good correspondence was also observed for a subset of quadrotor, SoDAR, and interpolated wind profiles corresponding to a period of moderate wind variability between 17:31 and 17:55 EDT (see Figure A6a,b in Appendix B). Alternatively, during periods of high wind variability, comparisons of quadrotor, SoDAR, and interpolated wind profiles were less consistent as is shown in both Figure 8a and Figure A8. However, in spite of the varied results for short-period comparisons, Figure 7b,c shows quadrotor and SoDAR observation trends to match well at 60 and 110 m AGL over a five hour duration of field experiments.

**Figure A9:**
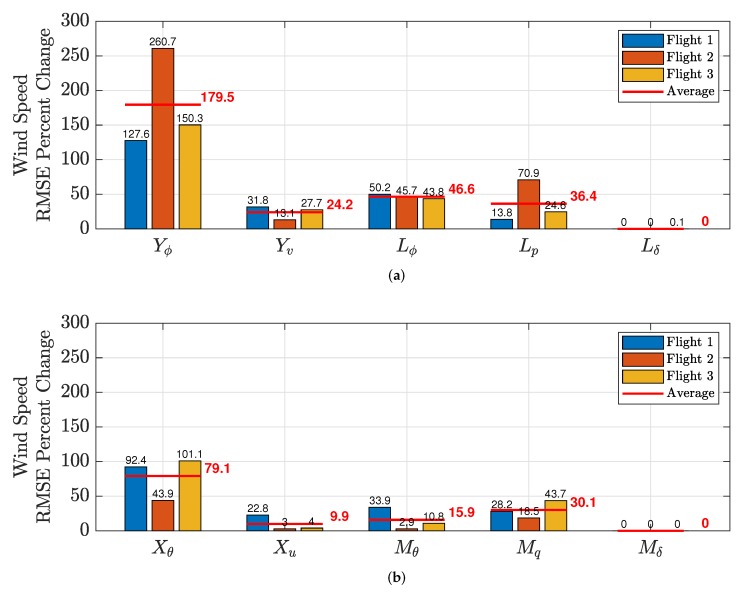
Sensitivity analysis of (**a**) roll and (**b**) pitch model parameters.

Following the validation of quadrotor wind profiles, a parameter sensitivity was conducted to assess how the accuracy of wind estimates degrades with parameter variations (see Appendix C). Model parameters were perturbed by the maximum difference between zeroth- and first-order parameter characterizations shown in Figure 5. Results from the sensitivity analysis show a strong to moderate dependence between the accuracy wind estimates and the parameters $Y_{\phi }$, $X_{\theta }$, $Y_{v}$, $X_{u}$, $L_{\phi }$, $M_{\theta }$, $L_{p}$, and $M_{q}$. As shown in Figure A9, there was a considerable percent change in wind estimation RMSE values when this subset of model parameters was perturbed. This outcome suggests that the accuracy of quadrotor wind estimates will decrease significantly when quadrotor operations deviate from the operating conditions for which the dynamic models have been characterized.

## 5. Discussion

Five models were identified to characterize the control-augmented rigid body dynamics of a quadrotor for wind estimation in hovering and steady vertical-ascent flight. An observability analysis confirmed that it is possible to estimate wind velocity using all five models. However, model parameter estimates were found to fluctuate significantly at higher ascent rates, which can greatly impact wind estimation error based on the sensitivity analysis presented in Appendix C. Parameter fluctuations, as mentioned in Section 4.1.2, may be the product of ambient flow and vehicle interactions at specific flight regimes or related to limitations with system identification experiments. Hence, more in-depth studies are required to understand the nature of parameter fluctuations at higher rates.

Anomalies were also detected in SoDAR wind measurements coinciding with periods of quadrotor operations. A two-part evaluation was performed to determine the nature of factors corrupting SoDAR observations. Examination of GPS position coordinates demonstrated the quadrotor to ascend through the sampling volume of SoDARs at approximately 60 m AGL. An assessment described in Appendix A of both the noise intensity and signal-to-noise ratio recorded by each SoDAR revealed a correlation between corrupted measurements and flight operations that strengthened with altitude. For this reason, it has been determined that quadrotor operations can significantly impact SoDAR observations when operating within the SoDAR’s sampling volume. Therefore, experiments involving SoDAR and multirotor operations in close proximity will have to mitigate for quadrotor noise.

A separate quadrotor with a small sonic anemometer on board was also considered as an alternative to validate model-based wind estimates. However, due to a hardware malfunction, the quadrotor-based wind sensor was not used in validation experiments. Other commercially available options employing indirect black-box methods for wind sensing, like the ones built into DJI multirotor aircraft, were not considered to validate model-based wind estimation. These alternatives are largely proprietary, do not offer wind data storage, and lack accuracy specifications for wind measurements. Thus, validation experiments were conducted for hovering and steady vertical-ascent flight wind estimates employing anemometer and SoDAR measurements only.

In spite of challenges with validation experiments, a considerable number of wind estimates were validated successfully. Wind estimates from the quadrotor hovering at 10 m AGL demonstrated good agreement between sonic anemometer and SoDAR measurements across all three flights. These results were found to be comparable to rigid-body model wind estimates reported in [1]. Thus, the rigid-body model wind estimation algorithms we use for measuring wind in hovering flight performs well across different quadrotor platforms. Quadrotor wind profile estimates, on the other hand, were validated using SoDAR observations from 10 to 120 AGL. Comparison results for periods of low wind variability demonstrate quadrotor wind profile estimates in close agreement with SoDAR wind speed and wind direction observations. This outcome provides impetus for additional comparisons to assess more closely the accuracy of model-based wind profiling inside the ABL.

Future work will involve improving validation experiments for a more thorough performance assessment of quadrotor wind profile estimates. Field experiments for wind estimate validation will require increasing the spatial separation between SoDARs to ensure quadrotor operations do not interfere with wind field measurements. Validation of model-based wind estimates will also incorporate in situ measurements from a sonic anemometer on board a separate quadrotor. Lastly, because coincident measurements are not feasible, validation experiments will have to take place when atmospheric conditions are relatively homogeneous and stationary, and significant uniformity of the wind field sampled by atmospheric sensors is expected.

## 6. Conclusions

An off-the-shelf quadrotor can be used to obtain model-based wind velocity estimates as long as the motion data logged on board the autopilot is accessible to the user. However, the accuracy of wind velocity estimates depends on how well the motion model characterizes the dynamics of the quadrotor for its operating condition. This paper extends a model based framework exploiting the rigid body dynamics of a quadrotor for hovering-flight wind estimation to estimate wind velocity along a vertical path in the lower atmosphere. The extension involved characterizing rigid body models for equilibrium flight conditions corresponding to each of five steady-ascending rates: ${V_{z}}_{\mathrm{eq}}=\left\{0.0,0.5,1.0,1.5,2.0\right\}$ m/s. Each quadrotor model was characterized employing stepwise regression and output error parameter estimation. An observability analysis confirmed the feasibility of estimating wind velocity using the identified model structures. Trends in parameter estimates also suggest that slower ascent rates may result in more accurate wind estimates. Significant variations in parameter estimates for higher ascent rates can be the outcome of limitations generating manually the rich and precisely timed excitation signals needed for model identification. Further studies are required to investigate this possibility in depth.

Field experiments were conducted to validate quadrotor wind estimates using in-situ and remote-sensing atmospheric sensors. Results from validation experiments demonstrated quadrotor wind estimates in hovering flight to be within within small error of sonic anemometer and SoDAR wind observations. Quadrotor wind profile estimates, on the other hand, were difficult to validate comprehensively because quadrotor operations affect the reliability of SoDAR wind measurements. However, in instances when atmospheric conditions were relatively invariant prior to and after quadrotor operations, quadrotor wind estimates demonstrated very good agreement with wind speed and wind direction from SoDAR measurements. Overall, this study demonstrates the feasibility of model-based vertical wind profiling using multirotor UAS in the lower atmosphere.

## Figures and Tables

**Table 1:** State measurements from autopilot’s AHRS.

State Measurement	Sate Variables	Sensor Type and Sampling Rate			
		Direct		Indirect	
Position	{x,y,z}	GPS	5 Hz	Barometer	8 Hz
				Extended Kalman Filter	8 Hz
Attitude	{ϕ,θ,ψ}	—	—	Gyroscope	18 Hz
				Accelerometer	18 Hz
				Extended Kalman Filter	8 Hz
Translational	{u,v,w}	GPS	5 Hz	Accelerometer	18 Hz
Velocity				Extended Kalman Filter	8 Hz
Angular Velocity	{p,q,r}	Gyroscope	18 Hz	—	—

**Table 2:** Accuracy specifications for sonic anemometer and SoDAR wind profilers.

Make/Model	Descriptor	Range	Resolution		Accuracy	
			Spatial	Temporal	Wind Speed	Wind Direction
ASC 4000i	LR-SoDAR	30–410 m	5 m	30 s	<0.5 m/s above 2 m/s	$2^{\circ }$ above 2 m/s
Remtech PA-0	SR-SoDAR	10–200 m	10 m	300 s	<0.2 m/s above 6 m/s	$3^{\circ }$ above 2 m/s
Gill MaxiMet GMX541	SA	N/A	N/A	1 s	3% at 12 m/s	$3^{\circ }$ at 12 m/s

**Table 3:** The plunge, yaw, roll and pitch model structures of the quadrotor determined from system identification flight experiments and step-wise regression algorithm presented in [51].

Model	Parameter Structure
Plunge	$\left(\begin{array}{c} \dot{z}\\ \dot{w} \end{array}\right)=\left(\begin{array}{cc} 0 & 1\\ 0 & Z_{w} \end{array}\right)\left(\begin{array}{c} z\\ w \end{array}\right)+\left(\begin{array}{c} 0\\ Z_{\delta } \end{array}\right)\delta_{\mathrm{plunge}}$
Yaw	$\left(\begin{array}{c} \dot{\psi }\\ \dot{r} \end{array}\right)=\left(\begin{array}{cc} 0 & 1\\ N_{\psi } & N_{r} \end{array}\right)\left(\begin{array}{c} \psi \\ r \end{array}\right)+\left(\begin{array}{c} 0\\ N_{\delta } \end{array}\right)\delta_{\mathrm{yaw}}$
Roll	$\left(\begin{array}{c} \dot{y}\\ \dot{\phi }\\ \dot{v}\\ \dot{p} \end{array}\right)=\left(\begin{array}{cccc} 0 & 0 & 1 & 0\\ 0 & 0 & 0 & 1\\ 0 & Y_{\phi } & Y_{v} & 0\\ 0 & L_{\phi } & 0 & L_{p} \end{array}\right)\left(\begin{array}{c} y\\ \phi \\ v\\ p \end{array}\right)+\left(\begin{array}{c} 0\\ 0\\ 0\\ L_{\delta } \end{array}\right)\delta_{\mathrm{roll}}$
Pitch	$\left(\begin{array}{c} \dot{x}\\ \dot{\theta }\\ \dot{u}\\ \dot{q} \end{array}\right)=\left(\begin{array}{cccc} 0 & 0 & 1 & 0\\ 0 & 0 & 0 & 1\\ 0 & X_{\theta } & X_{u} & 0\\ 0 & M_{\theta } & 0 & M_{q} \end{array}\right)\left(\begin{array}{c} x\\ \theta \\ u\\ q \end{array}\right)+\left(\begin{array}{c} 0\\ 0\\ 0\\ M_{\delta } \end{array}\right)\delta_{\mathrm{pitch}}$

**Table 4:** Nominal plunge and yaw model parameter estimates.

Speed	Plunge Model				Yaw Model			
	Parameter	Value	SE	Units	Parameter	Value	SE	Units
0–2 m/s	$Z_{w}$	−0.55	0.28	1/s	$N_{\psi }$	−1.71	0.41	$1/s^{2}$
	$Z_{\delta }$	−1.71	0.79	1/kg	$N_{r}$	−0.84	0.53	1/s
	–	–	–	–	$N_{\delta }$	2.41	1.18	1/($\mathrm{kg}\cdot m^{2}$)

**Table 5:** Nominal roll model parameter estimates.

Pitch Model	0.0 m/s		0.5 m/s		1.0 m/s		1.5 m/s		2.0 m/s		Units
Parameters	Value	SE	Value	SE	Value	SE	Value	SE	Value	SE	
$Y_{\phi }$	3.28	0.37	2.91	0.34	4.73	0.87	4.68	0.21	6.62	0.63	$m/s^{2}$
$Y_{v}$	−0.49	0.68	−0.31	0.04	−0.70	2.33	−0.62	0.14	−1.06	0.25	1/s
$L_{\phi }$	−4.54	4.17	−3.95	0.12	−5.87	2.55	−4.07	0.26	−5.92	0.10	$1/s^{2}$
$L_{p}$	−1.09	2.62	−1.15	0.22	−1.62	1.99	−0.82	0.23	−1.80	1.17	1/s
$L_{\delta }$	4.62	3.55	5.76	0.32	8.52	2.28	6.27	0.31	9.68	0.65	1/($\mathrm{kg}\cdot m^{2}$)

**Table 6:** Nominal pitch model parameter estimates.

Pitch Model	0.0 m/s		0.5 m/s		1.0 m/s		1.5 m/s		2.0 m/s		Units
Parameters	Value	SE	Value	SE	Value	SE	Value	SE	Value	SE	
$X_{\theta }$	−4.03	0.10	−3.94	0.12	−6.27	0.78	−5.48	0.14	−8.02	0.68	$m/s^{2}$
$X_{u}$	−0.71	0.56	−0.61	0.08	−0.80	0.19	−0.67	0.08	−1.24	0.28	1/s
$M_{\theta }$	−6.23	1.67	−5.20	0.11	−8.63	2.64	−4.44	0.23	−7.78	2.69	$1/s^{2}$
$M_{q}$	−1.46	0.87	−1.42	0.35	−2.63	0.65	−1.27	0.50	−2.09	0.84	1/s
$M_{\delta }$	6.61	0.36	6.32	0.28	10.80	1.98	6.81	0.40	10.70	0.64	1/($\mathrm{kg}\cdot m^{2}$)

**Table 7:** System identification validation results for plunge, yaw, roll and pitch models.

Ascent Rate	Plunge Model			Yaw Model			Roll Model			Pitch Model		
	Par.	RMSE	Units	Par.	RMSE	Units	Par.	RMSE	Units	Par.	RMSE	Units
0 m/s	*w*	0.44	m/s	*r*	2.59	rad/s	*v*	0.23	m/s	*u*	0.12	m/s
							*p*	0.39	rad/s	*q*	0.19	rad/s
0.5 m/s	*w*	0.44	m/s	*r*	2.59	rad/s	*v*	0.31	m/s	*u*	0.59	m/s
							*p*	0.21	rad/s	*q*	0.31	rad/s
1.0 m/s	*w*	0.44	m/s	*r*	2.59	rad/s	*v*	0.73	m/s	*u*	0.38	m/s
							*p*	0.90	rad/s	*q*	0.37	rad/s
1.5 m/s	*w*	0.44	m/s	*r*	2.59	rad/s	*v*	0.38	m/s	*u*	0.46	m/s
							*p*	0.48	rad/s	*q*	0.51	rad/s
2.0 m/s	*w*	0.44	m/s	*r*	2.59	rad/s	*v*	0.48	m/s	*u*	0.37	m/s
							*p*	0.71	rad/s	*q*	0.28	rad/s

**Table 8:** Comparison of wind speed and wind direction observations collected from the sonic anemometer and SR-SoDAR at 10 m AGL from 15:30 to 20:30 EDT on 5 June 2018.

Sensor	Height	Wind Speed			Wind Direction		
		Mean	MDE	RMSE	Mean	MDE	RMSE
SA	10 m	2.0 m/s	0.7 m/s	1.0 m/s	284${}^{\circ }$	32${}^{\circ }$	100${}^{\circ }$
SR-SoDAR		2.7 m/s			316${}^{\circ }$		

**Table 9:** Results from the comparison of SoDAR wind speed and wind direction observations collected from 15:00 to 20:30 EDT on 5 June 2018.

Sensor	Height	Wind Speed			Wind Direction		
		Mean	MDE	RMSE	Mean	MDE	RMSE
SR-SoDAR	60 m	4.5 m/s	−0.9 m/s	1.3 m/s	321${}^{\circ }$	−1${}^{\circ }$	25${}^{\circ }$
LR-SoDAR		3.6 m/s			320${}^{\circ }$		
SR-SoDAR	110 m	4.8 m/s	−0.9 m/s	1.4 m/s	321${}^{\circ }$	0${}^{\circ }$	26${}^{\circ }$
LR-SoDAR		3.9 m/s			321${}^{\circ }$		

**Table 10:** Comparison of wind speed and wind direction observations from the quadrotor, sonic anemometer, and SR-SoDAR collected at 10 m AGL between 18:05 to 20:17 EDT on 5 June 2018.

Flight Mode	Flight Time	Height	Wind Speed Mean Difference		Wind Direction Mean Difference	
			SA	SR-SoDAR	SA	SR-SoDAR
Hovering	18:05–18:15 EDT	10 m	0.9 m/s	0.5 m/s	$-8^{\circ }$	$-12^{\circ }$
			0.9 m/s	0.1 m/s	$12^{\circ }$	$1^{\circ }$
Hovering	18:19–18:27 EDT	10 m	−0.4 m/s	1.0 m/s	$25^{\circ }$	$17^{\circ }$
Hovering	20:08–20:17 EDT	10 m	−0.1 m/s	–	$-9^{\circ }$	–
**Absolute Mean Difference**			0.6 m/s	0.5 m/s	$14^{\circ }$	$10^{\circ }$

**Figure A1:**
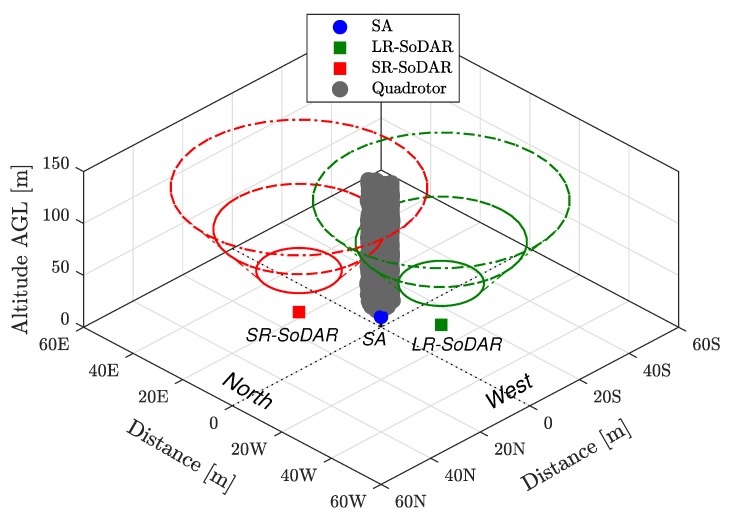
Spatial footprint of quadrotor operations relative to ground-based atmospheric sensors.

**Figure A2:**
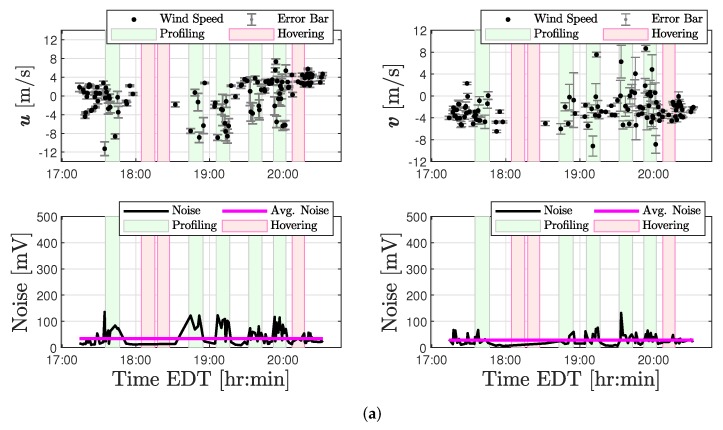

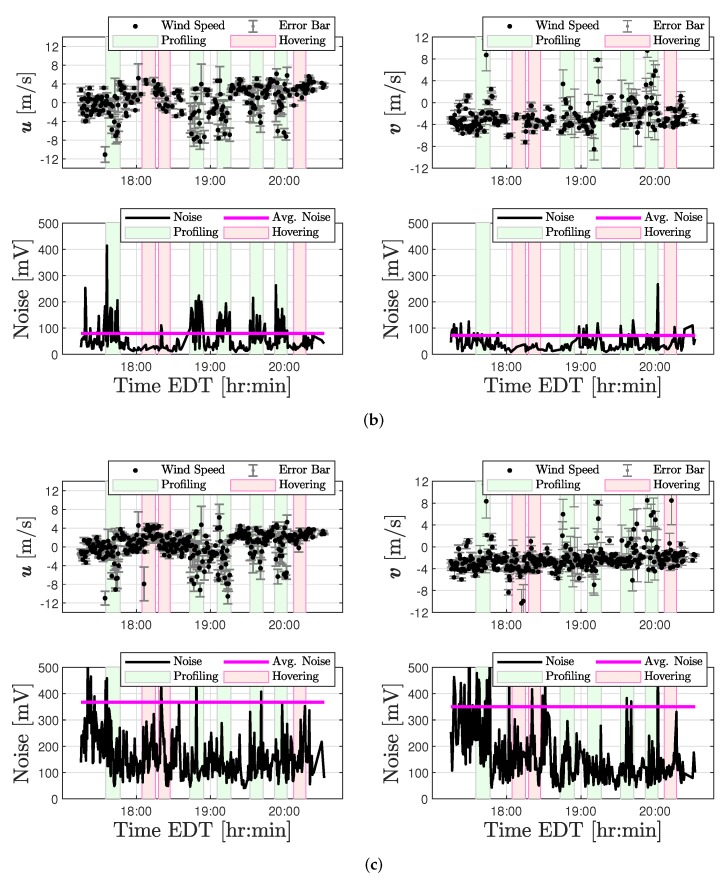
Noise intensity values for ***u*** and ***v*** wind velocity observations from the LR-SoDAR at (**a**) 110 m AGL, (**b**) 70 m AGL, and (**c**) 30 m AGL.

**Figure A3:**
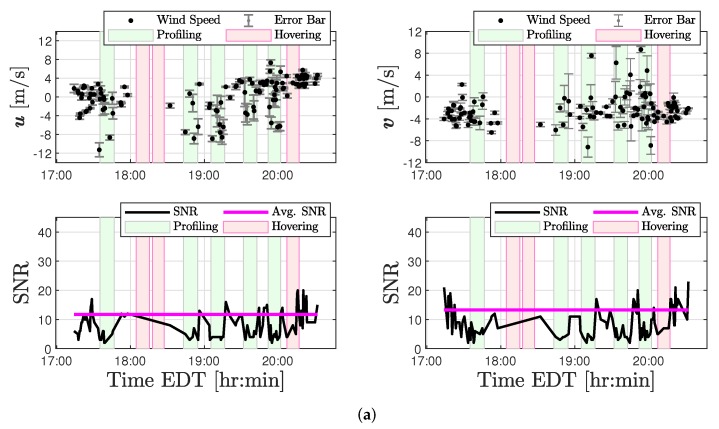

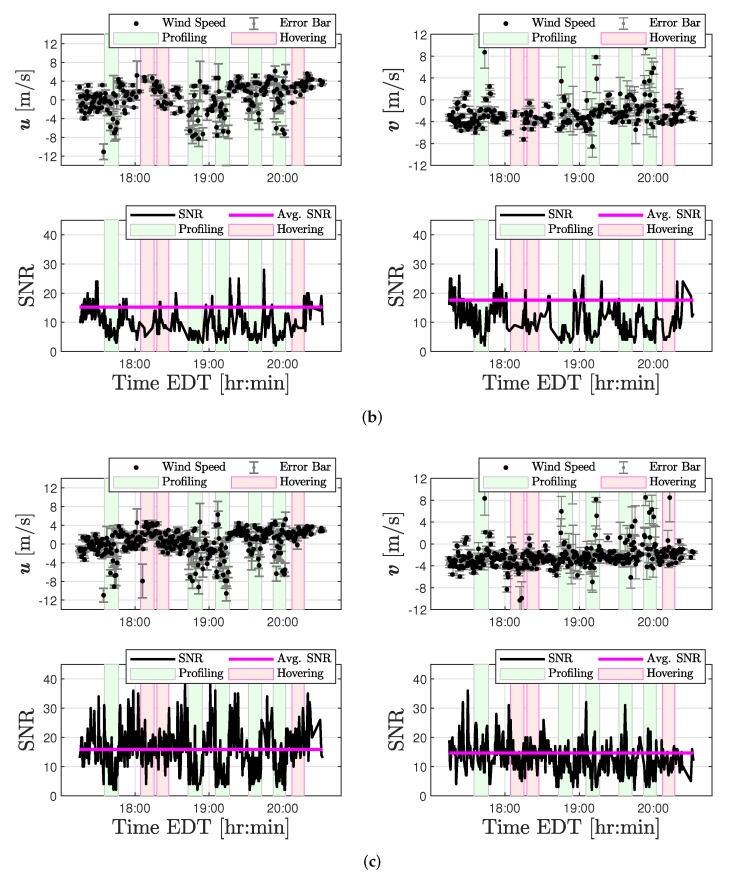
Singal-to-noise ratios for ***u*** and ***v*** wind velocity observations from the LR-SoDAR at (**a**) 110 m AGL, (**b**) 70 m AGL, and (**c**) 30 m AGL.

**Figure A4:**
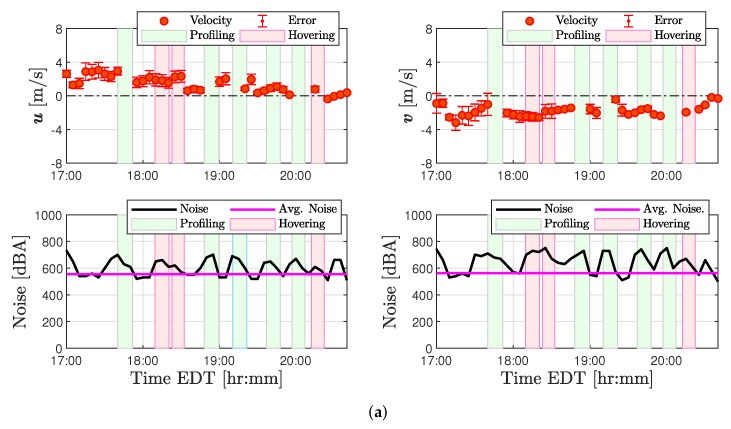

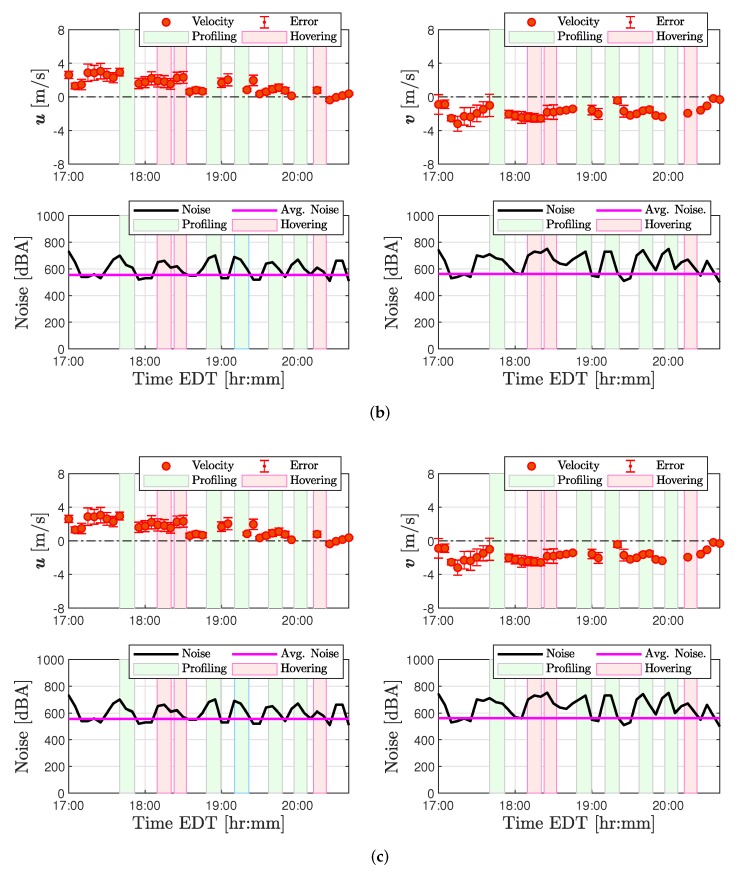
Noise intensity for ***u*** and ***v*** wind velocity observations from the SR-SoDAR at (**a**) 110 m AGL, (**b**) 70 m AGL, and (**c**) 30 m AGL.

**Figure A5:**
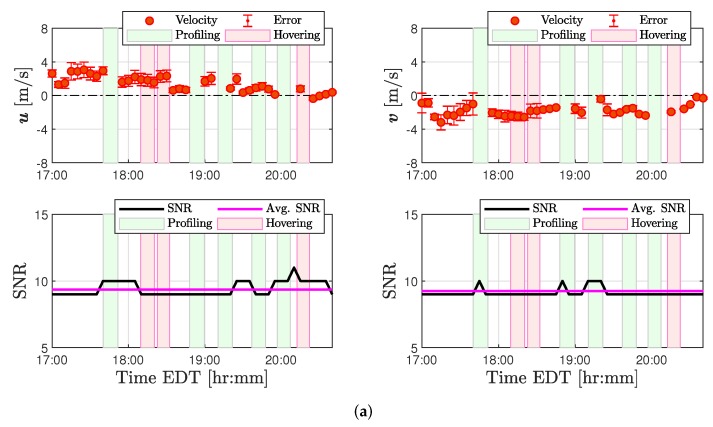

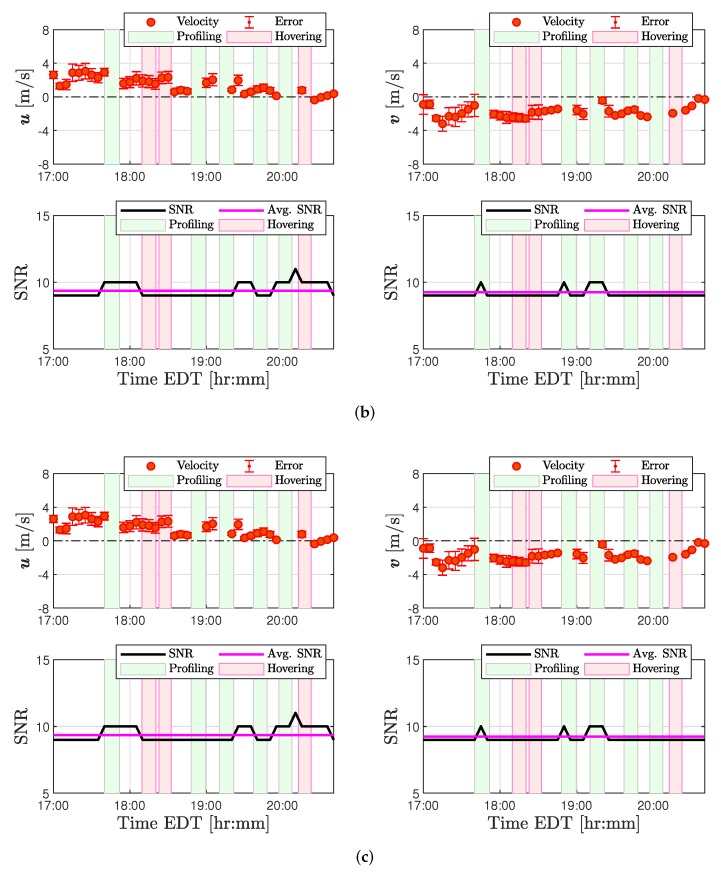
Signal-to-noise ratios for ***u*** and ***v*** wind velocity observations from the SR-SoDAR at (**a**) 110 m AGL, (**b**) 70 m AGL, and (**c**) 30 m AGL.

## Abbreviations

The following abbreviations are used in this manuscript:

ABL	Atmospheric boundary layer
AGL	Above ground level
AHRS	Attitude and heading reference system
Cov	Covariance
LR	Long range
MDE	Mean difference error
Par.	Parameter
PTH	Pressure, temperature, and relative humidity
RMSE	Root mean squared error
SA	Sonic Anemometer
SNR	Signal-to-noise ratio
SoDAR	Sound detection and ranging
SR	Short range
UAS	Unmanned aircraft system
ctrl	Control
ref	Reference

## Appendix A. A Reliability Study of SoDAR Wind Measurements

A two-part reliability study was conducted to investigate anomalies detected in SoDAR wind observations during quadrotor operations. The first part of the study looked at the spatial footprint of quadrotor operations relative to both the position and viewing angle of each SoDAR. The spatial footprint of quadrotor operations relative to SoDAR wind observations was determined from GPS position information provided by the quadrotor’s autopilot computer and SoDAR data logs. Quadrotor and SoDAR position information was used to determine if airframe obstruction of acoustic signals or propeller downwash corrupted SoDAR wind measurements. The second part of the study examined both the signal-to-noise-ratio (SNR) and noise intensity corresponding to wind measurements from each SoDAR prior to and during quadrotor operations. Combined, SNR and noise intensity SoDAR measurements were used to determine if anomalies in wind observations were attributed to quadrotor noise during flight operations. Findings from the two-part study can be used to inform best practices for integrating quadrotor and SoDAR operations for atmospheric wind sensing.

From the two-part reliability study it was determined that quadrotor flight operations impact SoDAR wind observations when operating in close proximity. Assessment of quadrotor’s flight path showed the quadrotor profiling through the sampling volumes of both the LR-SoDAR and SR-SoDAR 60 m AGL during flight operations. A 3-D rendering of this result is shown in Figure A1 where the ground position of the quadrotor and two SoDARs are plotted on the plot’s x-y plane relative sonic anemometer and the height of measurements is plotted on the z axis. Additionally, sudden changes in noise intensity and SNR during quadrotor operations were observed to coincide with corrupted wind measurements as shown in Figure A2, Figure A3, Figure A4 and Figure A5;. For example, u and v wind velocity components measured at 30, 70, and 110 m AGL with the LR-SoDAR show an abrupt increase in magnitude during quadrotor flights. Observations of u and v wind velocity components at 30, 70, and 110 m AGL were not logged by the SR-SoDAR during quadrotor operations. Both outcomes hinder the validation of quadrotor wind estimates at higher altitudes.

Results from the two-part study demonstrate a strong relationship between quadrotor noise and anomalies found in SoDAR wind observations. Based on our findings, quadrotor operations can interfere significantly with ground-based acoustic wind measurements. Precaution should be exercised when operating multirotor aircraft near SoDARs. Users will have to gauge a safe distance of separation based on the sampling volume of the SoDAR and the size of the multirotor aircraft used in flight operations.

## Appendix B. Quadrotor Wind Velocity Profiles

Additional quadrotor wind velocity profiles corresponding to constant vertical ascent rates of 1, 1.5 and 2 m/s are shown in Figure A6, Figure A7 and Figure A8.

## Appendix C. Sensitivity of Wind Estimates to Parameter Variations

Following the validation of quadrotor wind estimates from hovering flight, a sensitivity analysis was conducted to determine bounds on the accuracy of wind estimates resulting from parameter error. The nominal model used in the sensitivity analysis is characterized from the zeroth-order parameter values shown in Figure 5. The decrease in accuracy of wind estimates was quantified perturbing each zeroth-order parameter value at a time using the percent change of RMSE values as metric

(A1) $$\mathrm{RMSE}\;\mathrm{Percent}\;\mathrm{Change}=\frac{\left|\sqrt{\sum_{i=1}^{N}{\left[w-\hat{w}\right]}^{2}}-\sqrt{\sum_{i=1}^{N}{\left[w-{\hat{w}}^{*}\right]}^{2}}\right|}{\left|\sqrt{\sum_{i=1}^{N}{\left[w-\hat{w}\right]}^{2}}\right|}\times 100$$

where *w* is the true wind measurement, $\hat{w}$ is the unperturbed quadrotor wind estimates, ${\hat{w}}^{*}$ is the perturbed wind estimate, and *N* is the total number of wind estimates. The perturbation of each parameter was determined from the maximum difference between the zeroth- and first-order fit, which is shown in Figure 5 as well. Outcomes from this study are useful to understand the limitations of the nominal model for the range of steady-ascent rates that were employed for wind estimation.

Results from the sensitivity analysis confirms the accuracy of wind estimates diminishes as parameter error increases. As is shown in Figure A9, wind estimates degrade most significantly when when $Y_{\phi }$ and $X_{\theta }$ parameter values are varied. Parameter values $Y_{v}$, $X_{u}$, $L_{\phi }$, $M_{\theta }$, $L_{p}$, and $M_{q}$ also had a considerable effect on the accuracy of wind estimates. On the other hand, perturbations of $L_{\delta }$ and $M_{\delta }$ were observed to have no or insignificant impact. These results, combined, show that the use of a single model will lead to estimation errors when operating the quadrotor in off-nominal conditions.

## References

[1] González-Rocha J., Woolsey C.A., Sultan C., De Wekker S.F.J. (2019). Sensing wind from quadrotor motion. J. Guid. Control. Dyn..

[2] González-Rocha J., Woolsey C.A., Sultan C., De Wekker S.F. Model-based wind profiling in the lower atmosphere with multirotor UAS. Proceedings of the AIAA Scitech 2019 Forum.

[3] Barbieri L., Kral S.T., Bailey S.C.C., Frazier A.E., Jacob J.D., Reuder J., Brus D., Chilson P.B., Crick C., Detweiler C. (2019). Intercomparison of small unmanned aircraft system (sUAS) measurements for atmospheric science during the LAPSE-RATE campaign. Sensors.

[4] Jacob J., Chilson P., Houston A., Smith S. (2018). Considerations for atmospheric measurements with small unmanned aircraft systems. Atmosphere.

[5] Chilson P.B., Bell T.M., Brewster K.A., de Azevedo G.B.H., Carr F.H., Carson K., Doyle W., Fiebrich C.A., Greene B.R., Grimsley J.L. (2019). Moving towards a network of autonomous UAS atmospheric profiling stations for observations in the Earth’s lower atmosphere: The 3D mesonet oncept. Sensors.

[6] Smith S.W., Chilson P.B., Houston A.L., Jacob J.D. Catalyzing collaboration for multi-disciplinary UAS development with a flight campaign focused on meteorology and atmospheric physics. Proceedings of the AIAA Scitech 2017 Forum.

[7] Villa T., Gonzalez F., Miljievic B., Ristovski Z., Morawska L. (2016). An overview of small unmanned aerial vehicles for air quality measurements: Present applications and future prospectives. Sensors.

[8] Nolan P.J., Pinto J., González-Rocha J., Jensen A., Vezzi C., Bailey S., de Boer G., Diehl C., Laurence R., Powers C. (2018). Coordinated unmanned aircraft system (UAS) and ground-based weather measurements to predict Lagrangian coherent structures (LCSs). Sensors.

[9] Nolan P.J., McClelland H.G., Woolsey C.A., Ross S.D. (2019). A method for detecting atmospheric Lagrangian coherent structures using a single fixed-wind unmanned aircraft system. Sensors.

[10] Carranza V., Rafiq T., Frausto-Vicencio I., Hopkins F.M., Verhulst K.R., Rao P., Duren R.M., Miller C.E. (2018). Vista-LA: Mapping methane-emitting infrastructure in the Los Angeles megacity. Earth Syst. Sci. Data.

[11] Chao H., Chen Y. Surface wind profile measurement using multiple small unmanned aerial vehicles. Proceedings of the IEEE 2010 American Control Conference.

[12] Fairley P. (2018). Building a Weather-Smart Grid. Sci. Am..

[13] Phuangpornpitak N., Tia S. (2013). Opportunities and challenges of integrating renewable energy in smart grid system. Energy Procedia.

[14] Colak I., Fulli G., Bayhan S., Chondrogiannis S., Demirbas S. (2015). Critical aspects of wind energy systems in smart grid applications. Renew. Sustain. Energy Rev..

[15] Wildmann N., Bernard S., Bange J. (2017). Measuring the local wind field at an escarpment using small remotely-piloted aircraft. Renew. Energy.

[16] Varentsov M., Artamonov A.Y., Pashkin A., Repina I. (2019). Experience in the Quadcopter-Based Meteorological Observations in the Atmospheric Boundary Layer.

[17] Alsalous O., Galaviz R., Gulding J. Evaluation of the efficiency of traffic management initiatives wind delays. Proceedings of the 17th AIAA Aviation Technology, Integration, and Operations Conference.

[18] Tang W., Chan P.W., Haller G. (2010). Accurate extraction of Lagrangian coherent structures over finite domains with application to flight data analysis over Hong Kong International Airport. Chaos Interdiscip. J. Nonlinear Sci..

[19] Tang W., Chan P.W., Haller G. (2011). Lagrangian coherent structure analysis of terminal winds detected by lidar. Part I: Turbulence structures. J. Appl. Meteorol. Climatol..

[20] Knutson B., Tang W., Chan P.W. (2015). Lagrangian coherent structure analysis of terminal winds: Three-dimensionality, intramodel variations, and flight analyses. Adv. Meteorol..

[21] Rabinovich S., Curry R.E., Elkaim G.H. (2018). Toward dynamic monitoring and suppressing uncertainty in wildfire by multiple unmanned air vehicle system. J. Robot..

[22] Da Silva L.C.B., Bernardo R.M., de Oliveira H.A., Rosa P.F.F. Unmanned aircraft system coordination for persistent surveillance with different priorities. Proceedings of the 2017 IEEE 26th International Symposium on Industrial Electronics (ISIE).

[23] AL-Dhief F.T., Sabri N., Fouad S., Latiff N.A., Albader M.A.A. (2017). A review of forest fire surveillance technologies: Mobile ad-hoc network routing protocols perspective. J. King Saud Univ.-Comput. Inf. Sci..

[24] Xing Z., Zhang Y., Su C., Qu Y., Yu Z. Kalman filter-based wind estimation for forest fire monitoring with a quadrotor UAV. Proceedings of the 2019 IEEE Conference on Control Technology and Applications (CCTA).

[25] Duren R.M., Thorpe A.K., Foster K.T., Rafiq T., Hopkins F.M., Yadav V., Bue B.D., Thompson D.R., Conley S., Colombi N.K. (2019). California’s methane super-emitters. Nature.

[26] Hopkins F.M., Ehleringer J.R., Bush S.E., Duren R.M., Miller C.E., Lai C.T., Hsu Y.K., Carranza V., Randerson J.T. (2016). Mitigation of methane emissions in cities: How new measurements and partnerships can contribute to emissions reduction strategies. Earth’s Future.

[27] Smith B.J., John G., Christensen L.E., Chen Y. Fugitive methane leak detection using sUAS and miniature laser spectrometer payload: System, application and groundtruthing tests. Proceedings of the 2017 International Conference on Unmanned Aircraft Systems (ICUAS).

[28] Andersen T., Scheeren B., Peters W., Chen H. (2018). A UAV-based active AirCore system for measurements of greenhouse gases. Atmos. Meas. Tech..

[29] Juan Jesús R., Guillaume J., David S., Jaime del C., Antonio B. (2015). Mini-UAV based sensory system for measuring environmental variables in greenhouses. Sensors.

[30] Greene B.R., Segales A.R., Bell T.M., Pillar-Little E.A., Chilson P.B. (2019). Environmental and sensor integration influences on temperature measurements by rotary-wing unmanned aircraft systems. Sensors.

[31] Islam A., Houston A.L., Shankar A., Detweiler C. (2019). Design and evaluation of sensor housing for boundary layer profiling using multirotors. Sensors.

[32] Van den Kroonenberg A., Martin T., Buschmann M., Bange J., Vörsmann P. (2008). Measuring the wind vector using the autonomous mini aerial vehicle M2AV. J. Atmos. Ocean. Technol..

[33] Kocer G., Mansour M., Chokani N., Abhari R.S., Müller M. (2011). Full-scale wind turbine near-wake measurements using an instrumented uninhabited aerial vehicle. J. Sol. Energy Eng..

[34] Langelaan J.W., Alley N., Neidhoefer J. (2011). Wind field estimation for small unmanned aerial vehicles. J. Guid. Control. Dyn..

[35] McClelland H.G., Woolsey C.A. Effects of model simplification on wind reconstruction during open-loop longitudinal flight. Proceedings of the AIAA Scitech 2019 Forum.

[36] Elston J., Argrow B., Stachura M., Weibel D., Lawrence D., Pope D. (2015). Overview of small fixed-wing unmanned aircraft for meteorological sampling. J. Atmos. Ocean. Technol..

[37] Wolf C.A., Hardis R.P., Woodrum S.D., Galan R.S., Wichelt H.S., Metzger M.C., Bezzo N., Lewin G.C., de Wekker S.F. Wind data collection techniques on a multi-rotor platform. Proceedings of the IEEE Systems and Information Engineering Design Symposium (SIEDS).

[38] De Boisblanc I., Dodbele N., Kussmann L., Mukherji R., Chestnut D., Phelps S., Lewin G.C., de Wekker S. Designing a hexacopter for the collection of atmospheric flow data. Proceedings of the IEEE Systems and Information Engineering Design Symposium (SIEDS).

[39] Donnell G.W., Feight J.A., Lannan N., Jacob J.D. Wind characterization using onboard IMU of sUAS. Proceedings of the 2018 Atmospheric Flight Mechanics Conference.

[40] Hollenbeck D., Nunez G., Christensen L.E., Chen Y. Wind measurement and estimation with small unmanned aerial systems (suas) using on-board mini ultrasonic anemometers. Proceedings of the IEEE 2018 International Conference on Unmanned Aircraft Systems (ICUAS).

[41] Hollenbeck D., Oyama M., Garcia A., Chen Y. Pitch and roll effects of on-board wind measurements using sUAS. Proceedings of the 2019 International Conference on Unmanned Aircraft Systems (ICUAS).

[42] Prudden S., Fisher A., Mohamed A., Watkins S. A flying anemometer quadrotor: Part 1. Proceedings of the International Micro Air Vehicle Conference (IMAV 2016).

[43] Fuertes F.C., Wilhelm L., Porté-Agel F. (2019). Multirotor UAV-based platform for the measurement of atmospheric turbulence: Validation and signature detection of tip vortices of wind turbine blades. J. Atmos. Ocean. Technol..

[44] Neumann P.P., Bartholmai M. (2015). Real-time wind estimation on a micro unmanned aerial vehicle using its inertial measurement unit. Sens. Actuators A Phys..

[45] Brosy C., Krampf K., Zeeman M., Wolf B., Junkermann W., Schäfer K., Emeis S., Kunstmann H. (2017). Simultaneous multicopter-based air sampling and sensing of meteorological variables. Atmos. Meas. Tech..

[46] Palomaki R.T., Rose N.T., van den Bossche M., Sherman T.J., De Wekker S.F. (2017). Wind estimation in the lower atmosphere using multirotor aircraft. J. Atmos. Ocean. Technol..

[47] Wang J.Y., Luo B., Zeng M., Meng Q.H. (2018). A wind estimation method with an unmanned rotorcraft for environmental monitoring tasks. Sensors.

[48] Allison S., Bai H., Jayaraman B. (2020). Wind estimation using quadcopter motion: A machine learning approach. Aerosp. Sci. Technol..

[49] Tomić T., Schmid K., Lutz P., Mathers A., Haddadin S. The flying anemometer: Unified estimation of wind velocity from aerodynamic power and wrenches. Proceedings of the IEEE 2016 IEEE/RSJ International Conference on Intelligent Robots and Systems (IROS).

[50] Demitrit Y., Verling S., Stastny T., Melzer A., Siegwart R. Model-based wind estimation for a hovering VTOL tailsitter UAV. Proceedings of the 2017 IEEE International Conference on Robotics and Automation (ICRA), Marina Bay Sands.

[51] Klein V., Morelli E.A. (2006). Aircraft System Identification: Theory and Practice.

